# Acute disease induced cognitive dysfunction in older patients – an unrecognized syndrome

**DOI:** 10.1186/s12877-022-03323-w

**Published:** 2022-08-15

**Authors:** Rainer Wirth, Christiane Nicola Klimek, Gero Lueg, Maryam Pourhassan, Louisa Maria Danielzik, Caroline Krüger, Ulrike Sonja Trampisch

**Affiliations:** Department of Geriatric Medicine, Marien Hospital Herne, Ruhr University Bochum, Hölkeskampring 40, D- 44625 Herne, Germany

**Keywords:** Cognitive function, Delirium, Montreal cognitive assessment, Trajectories, Older

## Abstract

**Background:**

It is unknown, how many older hospitalized patients experience cognitive changes independently from delirium.

**Methods:**

In this retrospective study, cognitive function was assessed with the Montreal Cognitive Assessment on admission and discharge in 103 acute care geriatric hospital patients.

**Results:**

Mean age was 80.8 ± 7.3 years. The total MoCA score on admission was 17.8 (±4.5) and at discharge 17.7 (±4.4). The mean difference of the total MoCA score was − 0.1 (±3.5). 12 (11.7%) patients suffered from delirium. 46 (44.7%) patients experienced significant changes of cognitive function <− 2 or > 2 MoCA points without delirium. There was no significant association between delirium during hospital stay and the prevalence and magnitude of changes in total MoCA score.

**Conclusion:**

Cognitive changes frequently occur during acute disease of geriatric patients independently from delirium. We propose the term “acute disease induced cognitive dysfunction” (ADICD) for this entity.

**Trial registration:**

German Clinical trial register (DRKS-ID: DRKS00025157 on 28.04.2021).

## Background

The high prevalence of postoperative delirium and delirium during acute disease in older hospitalized patients is well known [1, 2]. However, in addition to the entity of delirium we frequently observe geriatric patients with changes in cognitive function during hospital stay without further clinical symptoms, i.e. not fulfilling the diagnostic criteria of delirium or subsyndromal delirium [3]. According to the literature, subsyndromal delirium seems to be as common as delirium [3, 4], and comprises the same diagnostic criteria as delirium, such as acute or subacute onset, disturbed attention, fluctuating course and disorganized thinking, but without the severity of delirium [5, 6].

For patients with cognitive dysfunction induced by a surgical intervention, the entity of postoperative cognitive dysfunction (POCD) has been described for this situation, which is distinct from postoperative delirium [7]. Surprisingly, this entity has not been characterized for patients without an operation.

However, in geriatric acute care patients with short-term follow-up measurement of cognitive function, we frequently observe significant changes in cognitive function without any other clinical symptom. Due to the lack of any related symptoms, we would not categorize this manifestation as subsyndromal delirium. In addition, because clinical symptoms are lacking, we assume that only in few patients these cognitive changes are detected during routine hospital care, in particular if a follow-up testing of cognitive function is not performed. Up to now, it is not known, how many older hospitalized patients experience cognitive changes independently from delirium and subsyndromal delirium. In addition, it is unclear with which magnitude these cognitive changes occur and if specific domains of cognitive function are affected more frequently.

Due to potential learning effects, cognitive follow-up testing is problematic and sometimes not reliable. Particularly cognitive screening instruments are mostly not validated for retesting [8]. However, the Montreal Cognitive Assessment (MoCA) [9], which is routinely used in the our geriatric hospital department, is validated for retesting while using three different forms [10]. Therefore, we analysed a set of randomly selected patients in which a MoCA-follow-up test was performed during one hospital stay, to measure the prevalence, magnitude and domains of cognitive changes during the acute care hospital stay of geriatric patients.

## Methods

### Study design and participants

This observational study was undertaken retrospectively between November 2018 to December 2019 in the acute care geriatric hospital department of Marien Hospital Herne, Ruhr University Bochum, Germany. Participants were admitted to the geriatric acute care ward. Inclusion criteria for the analysis were 60 years or older, and two screening results for cognitive function with the German MoCA on admission (MoCA original form) and at discharge (MoCA alternate-form). Exclusion criteria were palliative situation.

The study population comprised 103 older participants. Data were extracted retrospectively from medical records. Accordingly, we did not obtain informed consent from study subjects. The study protocol had been approved by the ethical committee of Ruhr University Bochum (no 19–6755-BR approved on 30.10.2020). The study is registered at German Clinical trial register (DRKS-ID: DRKS00025157 on 28.04.2021).

### Patients´ characteristics

Geriatric assessment was routinely performed at hospital admission including patients’ date of birth and sex. Participant’s age was calculated for the day of hospital admission. Years of education (≤ 12/> 12 years) were transferred from MoCA original form. Activities of daily living were determined using the Barthel-Index (BI) [11]. The point’s range for the German version of the BI is 0–100 pts., with 100 pts. indicating independence in all activities of daily living. Depressive symptoms were diagnosed using Depression in Old Age Scale (DIA-S) [12]. The point’s range for the German version of the 10-item scale is 0–10 pts., with 0–2 pts. indicating no, 3 pts. mild to moderate, and 4–10 pts. severe depressive symptoms. FRAIL scale [13] was used to identify persons at risk of frailty. The point’s range of the 5-item FRAIL scale is 0–5 pts., with 0 pts. indicating robust, 1–2 pts. pre-frail and 3–5 pts. frail functional status. SARC-F questionnaire [14] was used to identify persons at risk of sarcopenia. The point’s range for the German version of the 5-item SARC-F score is 0–10 pts., with 0–3 pts. indicating a low risk, and 4–10 pts. a high risk for sarcopenia. Hand grip strength was assessed with a hand dynamometer, and is defined as the maximum value from three attempts with the dominant hand privileged (if not possible, non-dominant hand). Risk of malnutrition was measured with the Mini Nutritional Assessment Short-Form (MNA-SF) [15]. The point’s range of the 6-item MNA-SF is 0–14 pts., categorized as normal nutritional status (12–14 pts.), at risk of malnutrition (8–11 pts.), and malnourished (0-7pts). The diagnosis of delirium during hospital stay (no/yes) and operation during the previous 14 day (no/yes) were taken from the patient’s medical record.

### MoCA

Cognitive function was assessed with the German MoCA [9]. The MoCA (original form) is part of the routine geriatric assessment during the admission procedure in the geriatric department since many years. The director of the department (RW) is a certified MoCA user. The point’s range of the MoCA is 0–30 pts., with higher pts. indicating normal cognitive function. The MoCA consists of seven cognitive domains including visuospatial (0–5 pts.), naming (0–3 pts.), attention (0–6 pts.), language (0–3 pts.), abstraction (0–2 pts.), delayed recall (0–5 pts.), and orientation (0–6 pts.). If the assessment with MoCA could not be fully recorded for non-cognitive reasons, i.e. in case of severe visual impairment, the result was extrapolated according to the amount of questions answered. In order to avoid any selection bias, the MoCA test was repeated with the alternate-form in the first patient on the discharge list 1 day before discharge from hospital [10]. If the MoCA had not been performed with this patient on admission due to rejection of the patient, the second patient on the list was tested. We used the MoCA alternate-form to reduce confounders related to repeated exposure [16]. The screenings were performed in a personal interview. Disturbances during cognitive testing were avoided by hanging a corresponding sign on the door. A trained and experienced occupational therapist performed the first screening with MoCA within 24 hours after hospital admission. It was carried out by up to three different therapists, depending on who was on duty on the ward at the day of admission. A psychologist (always the same person) performed the second screening with MoCA before discharge. The total MoCA score uncorrected for education was used for analyses.

### MoCA differences

In the present work, we calculated the differences between the total MoCA score from discharge and admission, and the differences in its seven cognitive domains (visuospatial, naming, attention, language, abstraction, delayed recall, and orientation). A difference of > 2 and < − 2 points was defined as a significant change in MoCA on an individual level [17].

According to the fact that cognitive changes could be detected during deterioration and recovery of cognitive function, the differences of the total MoCA scores are demonstrated as interval-scaled, and categorized in 3 groups (<− 2 pts./− 2 pts. to 2 pts./> + 2 pts.), and 2 groups (change/no changes (difference < − 2 pts. or > + 2 pts.)). A Bland-Altman plot was used to graphically demonstrate the consistency of the total MoCA scores. The Bland-Altman plot compares graphically the two (admission and discharge) total MoCA scores (values 0–30). It also shows possible relationships between measurement inaccuracies (estimated via the differences between the two total MoCA scores) with the level of the true value (estimated via the mean value of the two total MoCA scores) [18]. Therefore, the Bland-Altman plot allows identification of any systematic difference between the measurements or possible outliers. 95% limits of agreement for each comparison (average difference ± 1.96 standard deviation of the difference) were computed. The groups of significant change in MoCA (no change/change) were then linked to the presence or absence of delirium during hospital stay and operation during the last 14 days before admission to the geriatric department, and presented descriptively.

### Statistical analysis

The statistical analysis was completed using SPSS statistical software (SPSS Statistics for Windows, 137 IBM Corp, Version 27.0, Armonk, NY, USA). Means and standard deviations (SDs) were used for continuous data. Categorical variables are demonstrated as n (%). Chi Square tests were performed with delirium during hospital stay/operation during 14 days prior to admission and the prevalence of changes in total MoCA scores < − 2 or > + 2 points.

Statistical data analysis consisted of multiple logistic regression with change in total MoCA score < − 2 or > + 2 points (yes/no) as outcome variable. Analyses were performed using changes in total MoCA scores < − 2 or > + 2 points as reference. Possible confounder variables were selected a priori as the most important known causal and conditional risk factors, classified as either indicator variables (sex, delirium during hospital stay, operation during previous 14 days) or continuous variables (age: difference to mean), frailty, MNA-SF, MoCA score on admission). Corresponding odds ratios (OR, and their 95% CI) were calculated. Statistical significance was accepted at the two-sided 0.05 level, and all CI were computed at the 95% level.

## Results

### Characterization of study population

The study population comprised 103 older participants aged between 62 and 94 years (mean age 80.8 ± 7.3 years) with 61% being women. Characteristics of study population are shown in Table 1.

**Table 1:** Characteristics of the study participants

	n (%) or mean (sd)
Age in years at day of admission to hospital	80.8 (7.3)
Sex	
male	40 (38.8)
female	63 (61.2)
years of education	
> 12 years	63 (61.2)
≤12 years	40 (38.8)
Main treatment diagnosis	
osteoarthritis/fractures	34 (33)
cardiovascular	21 (20.4)
immobility/falls	21 (20.4)
stroke/neurodegenerative/delirium	14 (13.6)
Infection	5 (4.9)
Various	8 (7.8)
length of hospital stay (days)	15.5 (8.9)
Barthel-Index	48.4 (16.1)^a^
Depression in Old Age Scale (DIA-S)	3.1 (2.2)
no depressive symptoms (0–2 pts.)	47 (45.6)
mild to moderate depressive symptoms (3 pts.)	16 (15.5)
severe depressive symptoms (4–10 pts.)	40 (38.8)
Frail Scale	3.5 (1.1)
robust (0 pts.)	1 (1.0)
pre-frail (1–2 pts.)	21 (20.4)
frail (3–5 pts.)	81 (78.6)
SARC-F questionnaire	
low risk (0–3 pts.)	9 (8.7)
high risk (≥4 pts.)	94 (91.3)
Hand grip strength maximum value (kg)	15.5 (8.9)^a^
male	22.9 (6.8)^a^
female	10.7 (6.5)^a^
Mini Nutritional Assessment (MNA-SF)	
Normal nutritional status (12–14 pts.)	9 (9.3)
At risk of malnutrition (8–11 pts.)	48 (49.5)
Malnourished (0–7 pts.)	40 (41.2)
Delir during hospital stay	
no	91 (88.3)
yes	12 (11.7)
Operation previous 14 days	
yes	27 (26.2)
no	76 (73.8)

Data are n (%) or mean (standard deviation). ^a^ missing values in up to seven patients

### Total MoCA score and differences

**Fig. 1:**
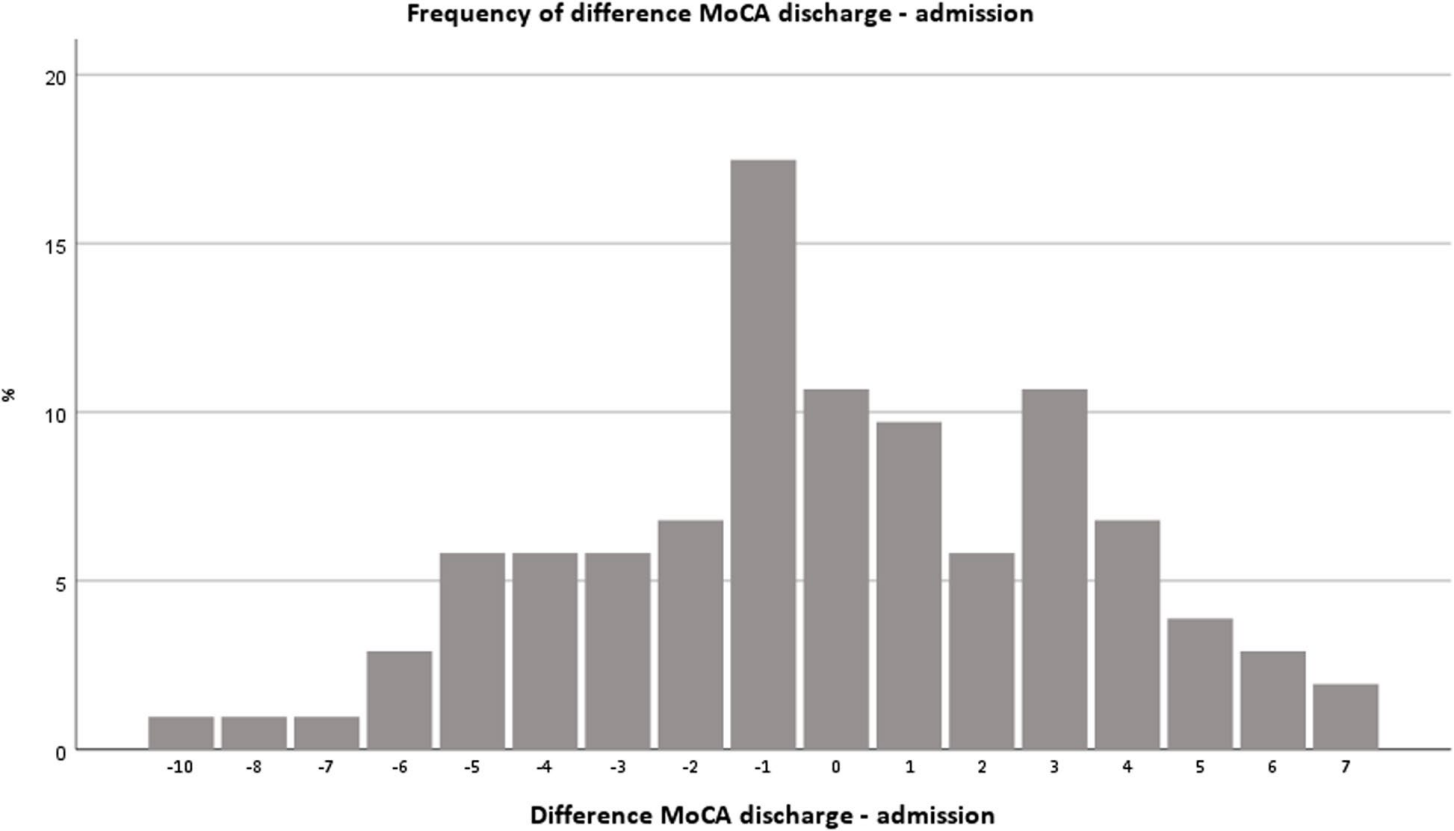
Frequency of difference of total MoCA scores discharge - admission

The total MoCA score on admission was 17.8 (±4.5) with a range from 5 to 27. The total MoCA score on discharge was 17.7 (±4.4) with a range from 6 to 26. The mean difference of the total MoCA scores (discharge - admission) was − 0.1 (±3.5) ranging from − 10 to 7. The highest frequency of differences was − 1 in 17.5% of patients (*n* = 18), followed by 0 with 10.7% (*n* = 11) and 3 with 10.7% of patients (see Fig. 1).

The results of MoCA’s seven domains (visuospatial, naming, attention, language, abstraction, delayed recall, and orientation) are shown in Table 2, separately for admission, discharge and the difference of both.

**Table 2:** Total MoCA score and domains separated by admission, discharge, and differences

	admission			discharge			difference^b^		
	mean (sd)	min	max	mean (sd)	min	max	mean (sd)	min	max
Total score	17.8 (4.5)	5	27	17.7 (4.4)	6	26	−0.1 (3.5)	−10	7
Visuospatial^a^	2.0 (1.3)	0	5	2.4 (1.2)	0	5	`0.4 (1.2)	−4	4
Naming^a^	2.8 (0.5)	1	3	2.5 (0.7)	0	3	−0.3 (0.7)	−3	1
Attention	4.4 (1.4)	0	6	4.6 (1.4)	1	6	`0.1 (1.4)	−3	4
Language	1.3 (0.9)	0	3	0.8 (0.9)	0	3	−0.5 (1.0)	−3	2
Abstraction	0.9 (0.7)	0	2	1.1 (0.8)	0	2	0.3 (0.9)	−2	2
Delayed Recall	1.3 (1.4)	0	5	1.1 (1.3)	0	5	−0.2 (1.5)	−5	4
Orientation	5.0 (1.4)	0	6	5.2 (1.3)	0	6	0.2 (1.1)	−3	4

*sd* Standard deviation. ^a^ missing values in 10 to 14 patients. ^b^ discharge-admission

Mean differences of the dimensions range from 0.4 (visuospatial) to − 0.5 (language). Regarding the differences in total MoCA score in patient categories, 27 (26.2%) patients had a difference of > 2, 52 (50.5%) a difference of − 2 to 2, and 24 (23.3%) a difference of <− 2. Consequently, 52 (50.5%) did not show any significant change in total MoCA scores from admission to discharge, whereas 51 (49.5%) showed a significant change with <− 2 or > 2. There was no significant association between delirium during hospital stay (*p* > 0.05) or operation during 14 days prior to admission (*p* > 0.05) and the prevalence of changes in total MoCA scores < − 2 or > 2 points (see Table 3). In addition, the absolute changes of MoCA-score in subjects with and without delirium did not differ significantly (data not shown).

**Table 3:** MoCA significant change and operation previous 14 days and delirium during hospital stay

	no change (*n* = 52)	change (*n* = 51)
operation previous 14 days		
no	35 (67.3)	41 (80.4)
yes	17 (32.7)	10 (19.6)
delirium during hospital stay		
no	45 (86.5)	46 (90.2)
yes	7 (13.5)	5 (9.8)

no change: total MoCA score discharge - admission = −2 to 2. change: total MoCA score discharge - admission = < − 2 or > 2. Data are n (%)

A review of the clinical situation of 10 patients with the most significant deterioration and improvement of cognitive function, respectively, revealed that a deterioration of cognitive function was associated with severe infection in two patients, decompensated heart failure in two patients, and delirium in one patient. However, in five patients an obvious clinical explanation for cognitive decline could not be revealed. Conversely, in patients with a significant improvement of cognitive function we found improvement of a pre-existing delirium in two patients, fading hyponatraemia in two patients, initiation of anti-dementia treatment in one patient and again no obvious clinical reason was found in five patients.

**Fig. 2:**
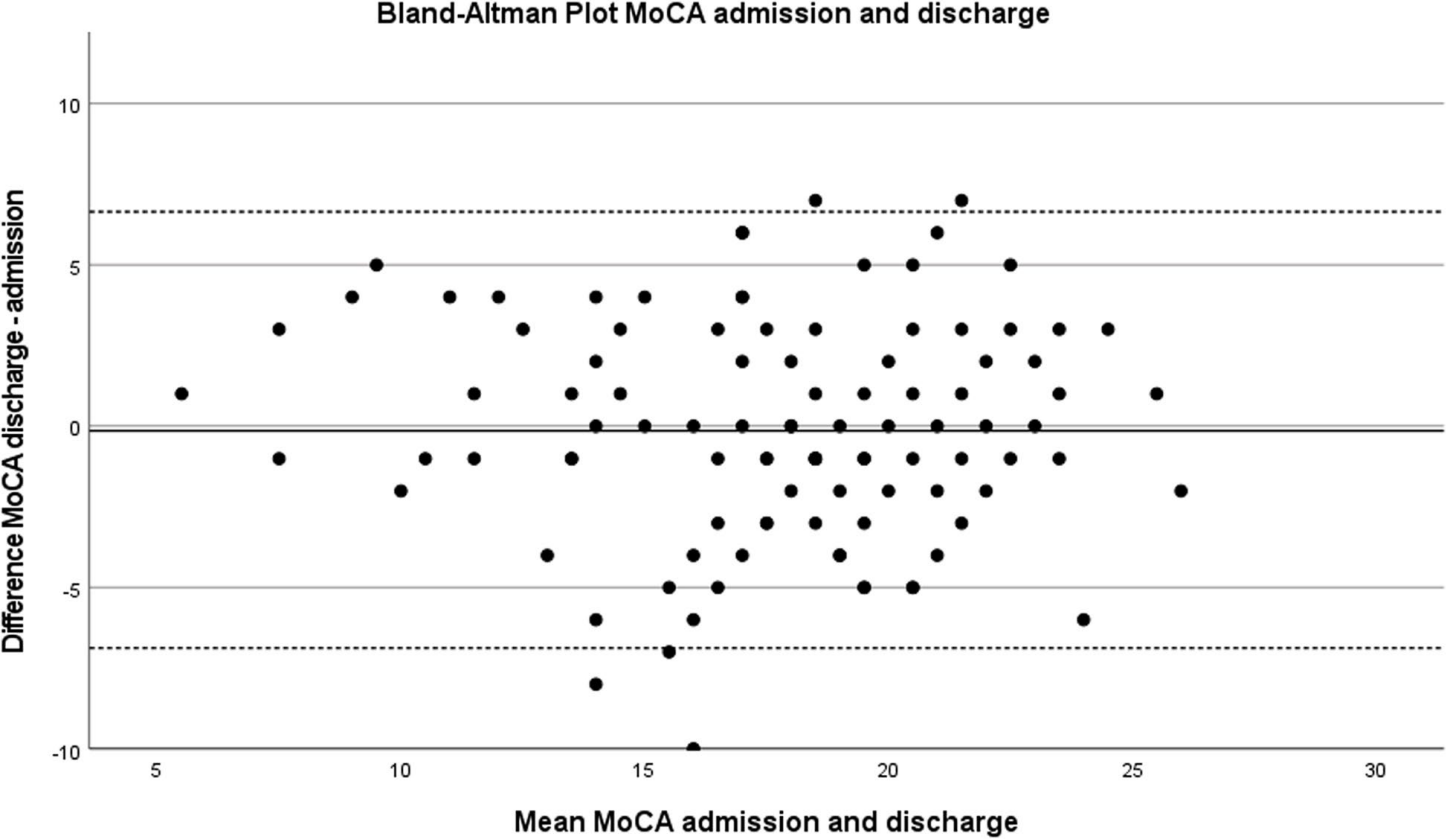
Bland-Altman Plot of agreement between total MoCA score admission and discharge

The 95% limits of agreement for the Bland-Altman plot were 6.6 (= (− 0.1 + 3.5)*1.96) and − 6.8 (= (− 0.1–3.5)*1.96). The Bland-Altman plot shows no systematic bias (e.g. higher range with increasing total MoCA score) between total difference and mean value of the two total MoCA scores (see Fig. 2). The mean difference (+ 0.1) between the two total MoCA scores indicates that there are minor deviations, visible from the solid line near 0 in the Bland-Altman plot. Measurements are equally distributed in between upper and lower limits of agreement, visible from the dashed lines.

### Multiple logistic regression

The multiple analyses did not show any significant associations. In the crude analyses, change in total MoCA scores were not associated with age (difference to mean) and male sex with odds ratios (OR) of 1.05 (95% CI: 0.99–1.11) and 1.45 (0.63–3.35), respectively. After adjustment for the covariates, estimates were slightly attenuated (Table 4). We did not find any significant associations.

**Table 4:** Odds ratios (with 95% confidence intervals) for changes in total MoCA scores < − 2 or > + 2 points

Variable	OR	95% CI
Age (difference to mean)	1.03	0.97–1.10
Male sex	1.34	0.52–3.48
Delirium during hospital stay	0.69	0.16–2.95
operation previous 14 days	0.63	0.24–1.65
Frailty	0.92	0.63–1.33
MNA-SF	0.99	0.82–1.18
MoCA score on admission	0.96	0.88–1.06

Multiple logistic regression analysis with changes in total MoCA scores < −2 or > + 2 points as dependent variables and all other variables as independent variables. *OR* Odds ratio, *CI* Confidence interval

## Discussion

In this cohort of 103 patients admitted to an acute care geriatric hospital ward, we found 12 (11.7%) patients with the diagnosis of delirium. However, 46 (44.7%) patients experienced significant changes of cognitive function <− 2 or > 2 MoCA points during hospital stay without delirium. Conversely, only half of the patients showed stable cognitive function during hospital stay, whereas one quarter demonstrated significant improvement and deterioration of cognitive function, respectively. The detected changes have not been pronounced in a specific domain of the MoCA test. Both directions of cognitive changes occurred, because geriatric patients frequently have been previously treated in other hospital departments demonstrating cognitive recovery during the actual stay in the geriatric department, implicating cognitive deterioration before admission to the geriatric ward. The results of the regression analyses support the working hypothesis that changes in MoCA scores appear regardless of the occurrence of a delirium, and any other reported variable. Altogether, there is a continuum of cognitive changes that occur during hospital stay of geriatric patients independently from delirium and subsyndromal delirium. The diagnosis of subsyndromal delirium includes, without the severity of delirium, the same diagnostic criteria as delirium, such as acute or subacute onset, disturbed attention, fluctuating course and disorganized thinking [5, 6]. The diagnosis of POCD includes a prior surgical procedure and is commonly referred to patients with persisting cognitive dysfunction [7, 19]. Most of the patients with cognitive changes in our study do not fulfil the criteria for either subsyndromal delirium or postoperative cognitive dysfunction. However, if a surgical procedure is capable of inducing acute cognitive deterioration independently from delirium [7], it would be self-evident that other severe stressors, such as acute disease without operation, are able to induce the same, particularly in vulnerable patients. So far, there is no concept for this entity. We therefore propose the term “acute disease induced cognitive dysfunction” (ADICD), analogue to POCD. Acute disease induced cognitive dysfunction could be defined as a measurable and significant cognitive decline with acute or subacute onset, associated with acute disease and presenting without symptoms of a delirium, such as disturbed attention, and disorganized thinking. Up to now there are no studies investigating short-term changes of cognitive function during acute disease or hospital stay of geriatric patients. This first pilot study is a description and analysis of the trajectories of cognitive function according to repeated MoCA during acute care hospitalization of geriatric patients. The results indicate frequent and relevant cognitive changes independently from delirium. The prevalence, magnitude and prognostic relevance of these changes should be evaluated in a prospective multicentre study to confirm the concept of “acute disease induced cognitive dysfunction” (ADICD).

This study has several limitations beside its single centre and retrospective design. The fact that delirium was not measured prospectively with a specific and validated tool is one disadvantage of the study. Furthermore, the prevalence of delirium is under representated, most likely due to the small sample size, but also because many patients with delirium are unable to participate in cognitive testing with MoCA.

Other additional variables (i.e. sensory impairment, history of alcohol use) that could have helped to explain these findings were unavailable and could not be included in the multiple analyses. In addition, the MoCA test was not performed by the same person on admission and discharge, which could have introduced an interrater bias. However, the duration of the cognitive changes and their prognostic relevance were not measured and remain thereby unclear. In general, the sample size is rather small, and results cannot be generalized. Further research to strengthen the results is needed.

## Conclusion

Cognitive changes frequently occur during acute disease of geriatric patients independently from delirium in our acute care geriatric hospital department. We propose the term acute disease induced cognitive dysfunction” (ADICD) for this entity.

## Data Availability

The datasets used and/or analysed during the current study are available from the corresponding author on reasonable request.

## Abbreviations

ADICD: Acute disease induced cognitive dysfunction
BI: Barthel-Index
DIA-S: Depression in Old Age Scale
MNA-SF: Mini Nutritional Assessment Short-Form
MoCA: Montreal Cognitive Assessment
POCD: Postoperative cognitive dysfunction
SD: Standard deviation
